# Management of ruptured ovarian teratoma mimicking advanced ovarian cancer

**DOI:** 10.1016/j.gore.2024.101386

**Published:** 2024-03-31

**Authors:** Katherine Jane Chua, Alice Barr, Miranda Prints, Rachel Ruskin, Rebecca Brooks

**Affiliations:** a University of California, Davis Medical Center, Department of Gynecology Oncology, USA; b University of California, San Francisco Fresno, Department of Obstetrics and Gynecology, USA

**Keywords:** Teratoma, Peritonitis, Ovarian cancer

## Abstract

• Chronic chemical peritonitis caused by spontaneous rupture of a mature cystic teratoma may result in prolonged hospitalization and respiratory decline and can mimic a gynecologic malignancy.
• Earlier surgical intervention for mature teratoma may prevent morbidity.
• Inclusion of a gynecologic oncologist is advised for management discussions and/or surgical back-up.
• Complex benign gynecologic surgeries may have some benefit for gynecologic oncologic trainees, which can be used for later oncologic cases.

## 1 Introduction

Mature teratomas are common benign neoplasms from which patients are typically asymptomatic (Suprasert et al., 2004). Spontaneous rupture is uncommon, reported to occur in less than one percent of cases (Suprasert et al., 2004, Bhatla et al., 1993). Rupture of mature teratomas may be acute and associated with chemical peritonitis rarely resulting in shock, or chronic with slow, continuous leakage of cyst contents (Suprasert et al., 2004, Bhatla et al., 1993). In cases of chronic rupture, patients may develop progressive abdominal distension, gastrointestinal disturbances, anorexia, and a granulomatous peritoneal reaction that may mimic advanced ovarian malignancy (Suprasert et al., 2004, Bhatla et al., 1993, Phupong et al., 2004).

We present a patient with a chronic ruptured mature teratoma, which mimicked an ovarian malignancy. Our case report contributes to the small number of reported cases of ruptured dermoid cysts and comments on the necessity of prompt identification and intervention to avoid surgical morbidity. We also explore the factors to be considered when selecting the appropriate specialty team to perform these surgeries.

## 2 Case presentation

**Fig. 1:**
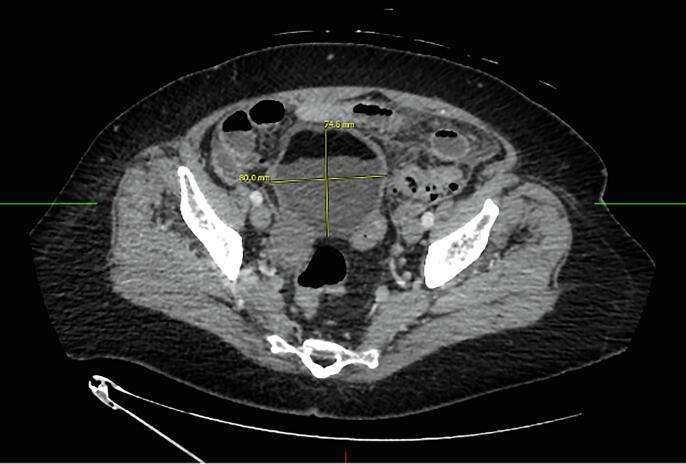
CT AP with contrast when patient initially presented to the hospital. 8.0 cm thick walled mass containing fat, fluid, soft tissue and calcification arising from the right ovary.

A 71 year-old female (Gravida 1 Para 0) with a 10-year history of a known 8 cm cyst presented with subacute abdominal pain for approximately three weeks. She was found to be anemic with a hemoglobin of 7.3 g/dl and malnourished (pre-albumin 5 mg/dL, albumin 2.2 g/dL) due to protein-calorie deficiency. The patient denied any vaginal bleeding or changes in bowel or urinary habits. Her initial computerized tomography (CT) scan demonstrated an 8 cm ovarian mass containing fat, fluid, soft tissue, and calcification as well as diffuse mesenteric thickening concerning for peritonitis (Fig. 1). She had elevated tumor markers with CA 125 of 70.1 U/mL and CEA of 43.3 ng/mL. CA 19–9 was normal at 3.3 U/mL. Her pelvic exam revealed a right adnexal mass consistent with imaging and tenderness on palpation. On admission, she was empirically treated for infectious peritonitis with intravenous ceftriaxone and metronidazole. Due to her elevated CEA, she underwent an esophagogastroduodenoscopy and colonoscopy, which showed no evidence of gastrointestinal malignancy. The general Gynecology service was consulted, and they recommended outpatient follow up.

**Fig. 2:**
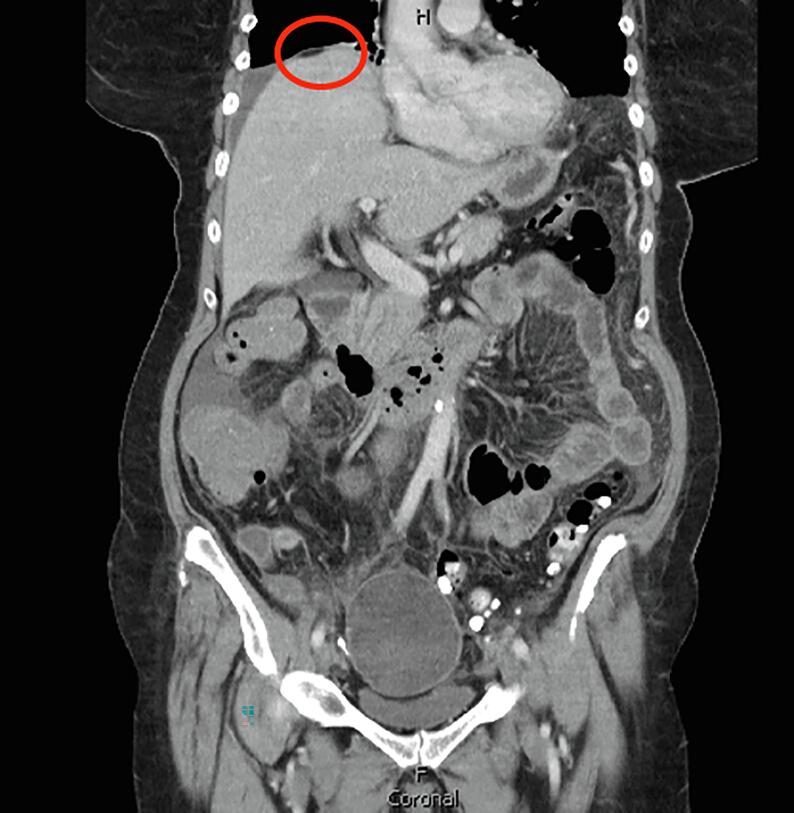
There is a new focus of fat under the right hemidiaphragm (circled in red), a new anterior cyst wall irregularity concerning for partial rupture, and ascites. (For interpretation of the references to colour in this figure legend, the reader is referred to the web version of this article.)

During the patient’s admission, she continued to develop worsening abdominal pain, ascites, and a new oxygen requirement. A repeat CT scan showed interval worsening of diffuse peritonitis with a new focus of fat under the right hemidiaphragm, a new anterior cyst wall irregularity concerning for partial rupture, and ascites (Fig. 2). The patient underwent a paracentesis, which did not show any malignant cells. Due to the adnexal mass with new ascites, the Gynecologic Oncology service was consulted due to suspected malignancy for surgical management. The patient developed worsening pain and an increasing oxygen requirement. After a multidisciplinary discussion, the decision was made to proceed with surgery to remove the suspected ruptured dermoid cyst as peritonitis from cyst rupture was suspected to be contributing to the patient’s respiratory symptoms.

**Fig. 3:**
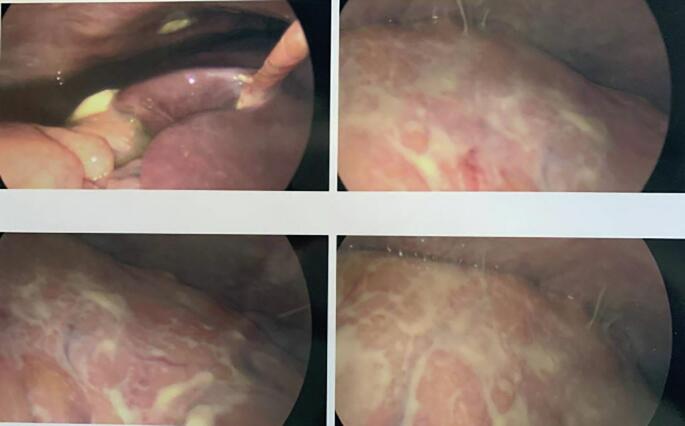
On diagnostic laparoscopy, there was profound infiltration of inflammatory change and exudate on the liver.

The patient underwent a diagnostic laparoscopy, which showed profound infiltration of inflammatory change and exudate on the omentum (Fig. 3). Multiple loops of small bowel were adherent to the anterior abdominal wall, omentum, and each other, precluding the ability to safely proceed with a minimally invasive approach. The case was converted to an exploratory laparotomy, where the entirety of the small bowel was seen matted to itself and to the omentum with yellow exudative plaque. The omentum was markedly inflamed (3–4 cm in thickness) without notable carcinomatosis. The right ovary was found to be twisted several times around its blood supply and grossly replaced by necrotic tissue. The right adnexal mass was sent for frozen pathology, which confirmed a necrotic mature cystic teratoma. The patient underwent a bilateral salpingo-oophorectomy, biopsy of the omentum and pericolic gutter, pelvic and abdominal lavage, and complex lysis of adhesions. Final pathology demonstrated a benign mature cystic teratoma without any immature elements.

During the patient’s post operative course, she developed a C. Difficile infection and a persistent oxygen requirement due to presumed atelectasis-induced hypoxia after a negative cardiopulmonary work-up. She otherwise recovered well from surgery and was discharged on post operative day (POD) #10. She returned to the hospital on POD#31 with nausea and abdominal distension initially concerning for a small bowel obstruction, which was ruled out. Her symptoms were thought to be secondary to an ileus from inflamed bowel caused by her chemical peritonitis. Her symptoms resolved with supportive care. The patient’s condition was improved 2 months post operatively, and subsequently was lost to follow up.

## 3 Discussion

Spontaneous rupture of mature cystic teratomas is extremely rare, occurring in 0.3–2 % of cases (Buzinkiene et al., 2019). Because the clinical signs of chemical peritonitis from rupture may mimic other disease processes such as gynecologic malignancy, gastrointestinal disease, or even pulmonary disease, multidisciplinary management and discussion may be warranted. Our patient had an elevated CEA with associated gastrointestinal symptoms, so she completed an EGD and colonoscopy and was ruled out for a gastrointestinal malignancy. A paracentesis was also performed due to her increasing ascites, which did not yield any malignant cells. However, a negative paracentesis does not rule out malignancy (Tejima et al., 2013). Additionally, due to her worsening respiratory status, the pulmonology team was consulted.

The recommended treatment for ruptured teratomas include surgical exploration with unilateral salpingo-oophorectomy and abdominal lavage. There is some reported benefit of proceeding with a minimally invasive approach for unruptured teratomas; however, there is concern for complications associated with spillage of the cyst contents. With laparotomy, there are decreased rates of clinical chemical peritonitis due to the ability to extensively irrigate (Wiberg et al., 2003). Removal of other pelvic organs including the uterus and the contralateral adnexa may be necessary depending on the extent of the damage caused from inflammation (Tejima et al., 2013, Stuart and Smith, 1983). The use of frozen section is important to reduce the risk of performing a more extensive surgery for a benign disease process (Tejima et al., 2013, Wiberg et al., 2003). At the time of surgery, it is not necessary to remove all visible implants (Phupong et al., 2004, Tejima et al., 2013, Wiberg et al., 2003, Stuart and Smith, 1983). For patients with significant inflammation, consideration of corticosteroid use may improve their post operative course (Suprasert et al., 2004, Wiberg et al., 2003).

Chemical peritonitis usually has a prolonged recovery course, often up to 1 year post operatively (Tejima et al., 2013). Patients who did not undergo surgical intervention experienced protracted recovery from their chemical peritonitis (Tejima et al., 2013). Earlier surgical intervention may result in decreased morbidity, prevention of new adhesions and nodules, and faster recovery for patients. Our patient had delays in her surgical management, which resulted in respiratory compromise and new oxygen requirements due to atelectasis.

Although our patient did not have final pathology consistent with a malignancy, malignant transformation should be considered during management of ruptured dermoid cysts. Malignant transformation of mature teratomas is a rare event with reported incidence of 0.2–2 % (da Silva et al., 2009, Koshiba, 2007, Hosokawa et al., 2010). The most common malignant transformations result in squamous cell carcinomas with risk factors including post-menopausal status (da Silva et al., 2009, Hosokawa et al., 2010). In cases where rupture has occurred, malignant transformation should be considered (Hosokawa et al., 2010). Gliomatosis peritonei (GP), more commonly associated with immature teratomas, are nodules see in the omentum or peritoneum and can be mistaken for carcinomatosis. GP is rarely associated with mature teratomas with case reports noted (Patel and Meena, 2022 Sep 6). Presence of GP may be useful to distinguish immature and mature teratomas. The utility of tumor markers in determining malignant transformation is unclear, with some studies showing a relationship between elevated SCC-antigen and CEA (Hackethal et al., 2008). An elevated CA-125 has not been shown to be a specific marker, given that any intraperitoneal inflammation may result in false elevation (Suprasert et al., 2004, Hackethal et al., 2008) as in the case of our patient.

The rarity of these cases, and the often unclear clinical picture, raises the question of which specialty team is best equipped to manage ruptured dermoid cysts. As mature cystic teratomas are benign, the management of rupture does not require surgical staging, and gynecologic oncologists may not always need to be involved. However, if there is complex peritonitis or clinical features concerning for malignant transformation such as elevated SCC-antigen or CEA, mass larger than 10 cm, or postmenopausal status, the inclusion of a gynecologic oncologist in the care team is warranted. The complex surgical decision making and expertise in such cases aligns with the skills of a gynecologic oncologist.

Boitano and Modesitt indicate that gynecologic oncologists are performing more benign gynecological surgeries (Boitano and Modesitt, 2024 Feb). However, Nguyen, et al demonstrated that trainees are uncomfortable with performing radical surgeries (Nguyen et al., 2024). These complex, though benign, surgeries can expand the surgical skillset of trainees to improve their comfort and confidence in later oncological cases. This case demonstrates the value of the appropriate level of expertise, be it benign, and the importance of gynecologic oncologist involvement in complex benign surgeries for patients and trainees.

In conclusion, we present a case of rare chronic chemical peritonitis caused by spontaneous rupture of a mature cystic teratoma resulting in prolonged hospitalization and respiratory decline. The clinical features of such cases may mimic other disease processes, including gynecologic malignancy and gastrointestinal disease, and the inclusion of a gynecologic oncologist in management discussions or for surgical back-up may be advised.

Written informed consent was obtained from the patient for publication of this case report and accompanying images.

**Author contributions**

KC, AB, and MP collected data and contributed to the manuscript with KC being lead on the case report. RR and RB supervised conceptualization and manuscript production including and reviewing and editing the manuscript.

## CRediT authorship contribution statement

**Katherine Jane Chua:** Writing – review & editing, Writing – original draft, Investigation, Conceptualization. **Alice Barr:** Writing – review & editing, Writing – original draft. **Miranda Prints:** Resources, Methodology, Conceptualization. **Rachel Ruskin:** Writing – review & editing, Supervision, Project administration, Investigation, Conceptualization. **Rebecca Brooks:** Writing – review & editing, Supervision, Resources, Project administration, Conceptualization.

## Declaration of competing interest

The authors declare the following financial interests/personal relationships which may be considered as potential competing interests: The following declarations of interest is for author Rebecca Brooks, MD. Speakers bureau – AstraZeneca, Consultant – GSK, Honorarium – MedLogix, Honorarium – Hay Market Media, Honorarium – OncLive. The other listed authors have no disclosures.

## References

[b0005] Bhatla N., Khanna R., Bhargava V.L. (1993). Intraperitoneal rupture of benign cystic teratoma. Int. J. Gynaecol. Obstet..

[b0010] Boitano T.L., Modesitt S.C. (2024 Feb). The more things change, the more they stay the same: the necessity of ensuring gynecologic oncologists remain surgical experts in a changing healthcare environment. Gynecol. Oncol. Rep..

[b0015] Buzinkiene D., Mongirdas M., Mikenas S., Drasutiene G., Andreika L., Sakalauskaite I. (2019). Chemical peritonitis resulting from spontaneous rupture of a mature ovarian cytis teratoma: a case report. Acta Med. Litu..

[b0020] da Silva B.B., dos Santos A.R., Lopes-Costa P.V., Sousa-Junior E.C., Correa-Lima M.V., Pires C.G. (2009). Ovarian dermoid cyst with malignant transformation and rupture of the capsule associated with chemical peritonitis: a case report and literature review. Eur. J. Gynaecol. Oncol..

[b0025] Hackethal A., Brueggmann D., Bohlmann M., Franke F.E., Tinneberg H.-R., Munstedt K. (2008). Squamous-cell carcinoma in mature cystic teratoma of the ovary: a systematic review and analysis of published data. Lancet Oncol..

[b0030] Hosokawa T., Sato Y., Seki T., Maebara M., Ito K., Kuribayashi S. (2010). Malignant transformation of a mature cystic teratoma of the ovary with rupture. Jpn. J. Radiol..

[b0035] Koshiba H. (2007). Severe chemical peritonitis caused by spontaneous rupture of an ovarian mature cystic teratoma: a case report. J. Reprod. Med..

[b0040] Nguyen N.T., Jang A.M., Pomerantz T., Zavorsky G.S., Leiserowitz G., Brooks R.A. (2024). Perspectives of gynecologic oncology fellowship training and preparedness for practice. Gynecol. Oncol. Rep..

[b0045] Patel T., Meena V. (2022 Sep 6). Gliomatosis peritonei and its relation to teratoma: role of imaging and histological aspects. Cureus..

[b0050] Phupong V., Sueblinvong T., Triratanachat S. (2004). Ovarian teratoma with diffused peritoneal reactions mimicking advanced ovarian malignancy. Arch. Gynecol. Obstet..

[b0055] Stuart G.C., Smith J.P. (1983). Ruptured benign cystic teratomas mimicking gynecologic malignancy. Gynecol. Oncol..

[b0060] Suprasert P., Khunamornpong S., Siriaunkgul S., Phongnarisorn C., Siriaree S. (2004). Ruptured mature cystic teratomas mimicking advanced stage ovarian cancer: a report of 2 cases study. J. Med. Assoc. Thai..

[b0065] Tejima K., Enomoto R., Arano T. (2013). A case of chemical peritonitis and pleuritis caused by spontaneous rupture of a benign cystic ovarian teratoma that improved without surgical intervention. Clin. J. Gastroenterol..

[b0070] Wiberg N., Kiss K., Dalsgaard L. (2003). Lipogranuloma peritonealis caused by spontaneous rupture of a benign cystic ovarian teratoma. Acta Obstet. Gynecol. Scand..

